# The Roles of Primary cilia in Polycystic Kidney Disease

**DOI:** 10.3934/molsci.2013.1.27

**Published:** 2014

**Authors:** Sarmed H. Kathem, Ashraf M. Mohieldin, Surya M. Nauli

**Affiliations:** 1 College of Pharmacy and Pharmaceutical Sciences, University of Toledo, Toledo, Ohio; 2 College of Pharmacy, University of Baghdad, Baghdad, Iraq

**Keywords:** aneurysm, chemosensory, hypertension, renal epithelia, vascular endothelia

## Abstract

Autosomal dominant polycystic kidney disease (ADPKD) is an inherited genetic disorder that results in progressive renal cyst formation with ultimate loss of renal function and other systemic disorders. These systemic disorders include abnormalities in cardiovascular, portal, pancreatic and gastrointestinal systems. ADPKD is considered to be among the ciliopathy diseases due to the association with abnormal primary cilia function. In order to understand the full course of primary cilia and its association with ADPKD, the structure, functions and role of primary cilia have been meticulously investigated. As a result, the focus on primary cilia has emerged to support the vital roles of primary cilia in ADPKD. The primary cilia have been shown to have not only a mechanosensory function but also a chemosensory function. Both structural and functional defects in primary cilia result in cystic kidney disease and vascular hypertension. Thus, the mechanosenory and chemosensory functions will be analyzed in regards to ADPKD.

## 1. Introduction

Autosomal dominant polycystic kidney disease (ADPKD) is the most common inherited cystic renal disease and is considered the most common single gene disorder of the kidneys. ADPKD affects approximately 600,000 people in the United States and 1:400 to 1:1000 people worldwide [1,2]. ADPKD is a systemic disorder that includes a variety of renal and extra-renal abnormalities that ultimately result in cystic and non-cystic features. The main clinical characteristic of the disease, however, is the progressive increase in the number and size of renal cysts, with secondary destruction of renal parenchyma.

In a prospective clinical study, Grantham JJ et al showed that the total kidney volume in ADPKD patients increased from 204 ml to 218 ml over 3 years, which estimates the total cyst volume increase as 5.27% per year [3]. On the other hand, renal function deterioration was estimated as a decline in glomerular filtration rate of 4.3 ± 8.1 mL/min per year. This ultimately leads to end stage renal disease (ESRD) in about 43–48% of patients by 58–73 years of age [4].

In addition to renal cysts, renal manifestations include urinary tract infection, flank pain, hematuria, nephrolithiasis and renal failure [5,6,7]. Extra-renal cystic features are also developed in organs including the liver, pancreas, ovaries and choroid plexus. Cardiovascular abnormalities include vascular hypertension, left ventricular hypertrophy, intracranial aneurysms, aortic aneurysms, arachnoid aneurysms, cerebral artery dolichoectasia, mitral valve prolapse, mitral regurgitation, aortic insufficiency, and tricuspid regurgitation. Although renal characteristics are prominent features, the cardiovascular abnormalities are responsible for 80% more deaths in ADPKD than ESRD. Furthermore, intracranial aneurysms affect 4–12% of ADPKD patients, with a risk of rupture about five-fold more than in the general population. Thus, aneurysm rupture is considered a serious complication threatening the lives of ADPKD patients [6,8].

Most importantly, ADPKD is a pathology associated with cilia dysfunction, also known as ciliopathy [9,10]. The primary cilium is a solitary “9 + 0” microtubule-based, hair-like organelle anchored to the mother centriole and projecting from the surface of almost all mammalian cells. In addition to the wide range of sensory functions, primary cilia are also critical for developmental and physiological functions. Historically, the story of this cellular antenna is really interesting; it was first described by Zimmermann as early as 1898 [11]. Since that time, primary cilia were regarded as nonfunctional remnants from evolution. As a result, the research on primary cilia was relatively limited until the last decade, when extensive research has been focused on this organelle. Moreover, an assessment of the research done on primary cilia in the last five decades, using PubMed search, revealed that the research comprised only about 10% of the total research performed on primary cilia from 1960 to 2000. This means that primary cilia research increased nine-fold in the last decade compared to the previous four decades. Therefore, not surprisingly, the rapidly growing focus on primary cilia since the year of 2000 has attracted researchers’ interest to uncover many unknown entities and relate them to diseases associated with defective cilia structure/function.

**Figure 1:**
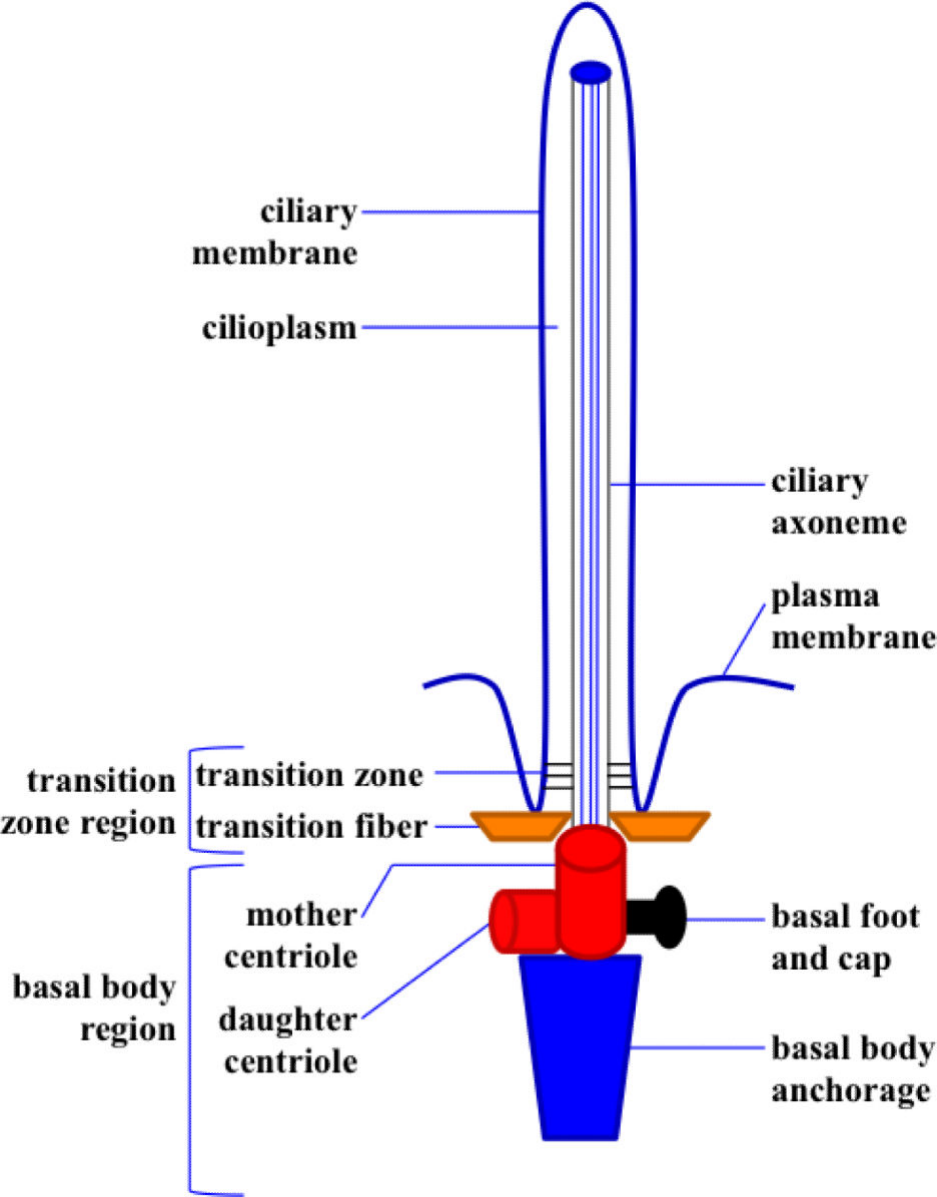
The primary cilium is composed of ciliary membrane, cilioplasm, axoneme and basal body. Basal bodies are composed of transition fiber (orange), centrioles (red), basal foot and cap (black) and basal body anchorage (blue). The ciliary membrane and axoneme make up the upper part of the primary cilia.

Structurally, the primary cilium composed of five main compartments [12] (Figure 1). The*axoneme* is composed of nine parallel pairs of microtubules posttranslationally acetylated to support the long structure. These microtubules are arranged circumferentially, without a central pair like the one that is always seen in motile cilia. The *ciliary membrane* houses many receptors, ion channels, transporters and sensory proteins that serve definitive functions. Many of these proteins are not yet completely established. Some of those receptors are localized to the ciliary membrane only at a certain time to perform a defined function and then translocated out of the cilia. *Cilioplasm* is constituted of the soluble compartment of the cilia. It has been recently proposed that cilioplasm acts as a calcium signaling compartment in response to mechanical and chemical stimuli [13]. Cilioplasm is also enriched with many other signaling proteins. Thus, this dynamic compartment includes mainly two types of proteins, signaling and transport (such as intraflagellar transport) proteins (like IFT proteins). Both signaling and transport proteins are required to coordinate a key role in cilia assembly and function. The *basal body* is a mother centriole to which the ciliary axoneme is rooted. In addition to its vital structural role, the basal body houses many signaling proteins that serve various functions. The *transition zone* region composes of transition zone and fibers. The region connects basal body and ciliary axoneme and plays critical roles in ciliogenesis and ciliary access [14].

## 2. Roles of Primary Cilia

Owing to the unique localization of a variety of receptors, ion channels, transport proteins and signaling proteins, primary cilia serve a broad range of functions. Recent ciliary genomics and proteomics data sets have estimated that the vertebrate cilium function might involve about 1000 different polypeptides [15]. Working as a cellular antenna, primary cilia sense and conduct a range of signaling pathways from mechanical and chemical stimuli [16,17,18]. The ciliary pathways studied include signaling through calcium, sonic hedgehog, Wnt, mTOR, JAK/STAT, and MAPK, among a growing list. These signaling pathways play a key role in various vital cellular processes like development, differentiation, cell cycle, apoptosis, tissue homeostasis and planar cell polarity [19].

Apart from playing a chemosensory role manifested as receiving extracellular information, primary cilia may also perform the opposite “chemosecretory” function, manifested as releasing information to the extracellular environment [20]. This new area of research is supported by a study revealing that polyductin, a ciliary membrane protein, undergoes proteolytic cleavage with the release of extracellular domain into the lumen [21]. In addition, polycystin-1 is shown to undergo cleavage with the secretion of a small amount of N-terminal domain to the extracellular environment [22]. Furthermore, membrane-sheathed objects carry Shh and retinoic acid secreted from the ciliated cells of the embryonic node in response to fluid flow, critical for left-right determination [23]. Equally important, many PKD-associated proteins form exosome-like vesicles, which are shed in the urine [24]. Exosomes are produced by the cell and released from the cell membrane. Because the exosomes emerge from an intracellular vesicle near the base of the cilium, the authors suggest that some exosomes proteins are derived from cilia. Furthermore, exosomes interact with and adhere to ciliary membrane. Although the shedding of these proteins has an unknown function, the idea of ciliary chemosensory function is interesting and worth further investigation.

## 3. Mechanosensory and Chemosensory Cilia Functions

Functioning as cellular antennae, primary cilia receive a complex pool of external stimuli and transduce them into intracellular signaling to control an expanding list of cellular functions. These external stimuli may consist of physical stresses like flow and pressure, or chemical substances like ligand, growth factor and morphogen. One of the most studied ciliary functions is mechanosensation, which is a flow sensing ability of the primary cilium to sense the overpassing fluid. Genetically manipulated non-ciliated cells or chemically ablated cilia from ciliated cells are found to be mechano-insensitive to fluid flow, supporting ciliary mechanosensory function [19,25]. It is generally accepted that polycystin-1 and -2 are two of many ciliary proteins responsible for the mechanosensing function attributed to the primary cilia [26,27,28]. Furthermore, the ciliary bending model in response to fluid dynamics, hypothesized by Schwartz *et al* [29], has gained more support through recent studies with different experimental designs [30,31]. In this model, the flexural rigidity of primary cilia is calculated to predict the cilium bending behavior, where a “heavy elastica” model is validated to interpret the mechanosensory function as a result of cilium bending. Recently, our laboratory further confirmed cilia bending-induced calcium signaling [13]. Our data show that cilium bending causes cytoskeletal deformation, and there is a lag time between bending, which is fast, and the delayed cytosolic calcium increase. Cilium bending results in stress building up on the cell membrane, caused by the stretching property of the membrane, which is delayed compared to bending. It is postulated that stress by bending is localized at the base of ciliary membrane, and the delay in calcium response upon cilia bending is caused by mechanical properties of the cell membrane [32]. Our laboratory recently shows that polycystin-2 channel opens to let calcium ions enter into the cilioplasm [13].

Looking at the substantial heterogeneity in flow chamber design, shear stress forces, cell types and other experimental varieties involved in the studies of ciliary mechanosensation, the data strongly conclude that the polycystin-1 and -2 complex localizes to the mechanosensory compartment of primary cilia [33]. Collectively, the ciliary bending model [29], bending-induced membrane stretching at the base of primary cilia [32], or any other hypothetical model to interpret mechanosensation [31] indicate that fluid flow can cause a conformational change within the ciliary membrane.

Another type of external stimuli received by the primary cilia is a chemical signal. The interaction of any chemical mediator or ligand to its specific receptor with the subsequent signaling cascade housed in the primary cilium renders this organelle as a chemosensor. Dopamine receptor type-5 [34], 5-HT6 receptor [35], somatostatin receptor-3 [36], purinergic P2Y_12_ receptor [37], melanin concentrating hormone receptor-1 [38], patched and smoothened receptors of hedgehog [39,40], Wnt signaling network [41], PDGFRα [42], and vasoactive intestinal receptor-2 [43], among others, are examples of receptors and their associated signaling cascades localized to the primary cilia [12]. An outstanding study from Christensen laboratory further shows that PDGFRα dimerizes and is phosphorylated in the cilium [44]. Our laboratory and others have further confirm the ciliary function in the process of wound healing [44,45,46]. Thus, the unique localization of these signaling pathways proposes the primary cilium as a chemosensor and a key coordinator of various cellular signaling and functions.

A recent study also elegantly shows that a functional ciliary complex composed of polycystin-2, adenylyl cyclase-5/6, phosphodiesterase-4C and A-kinase anchoring protein-150 are cross-talked in the primary cilia to regulate cAMP level [47]. In addition, another study suggests that Mchr1 and Sstr3 form heteromers in the primary cilia membrane, a process that modulates ligand binding properties as well as downstream signaling [48]. More recently, it was shown that ciliary localization of GRP88 protein plays an important role in negatively regulating ciliary D1 dopamine receptor function, while asserting its inhibitory effect on non-ciliary β2 adrenergic receptor [49]. These studies provide evidence of the functional cilia receptor interaction and open the way to further formulate the idea of a cilium as a centerpiece of receptors homing.

## 4. Renal Epithelial Function of Cilia

### 4.1 Mechanosensory primary cilia

**Figure 2:**
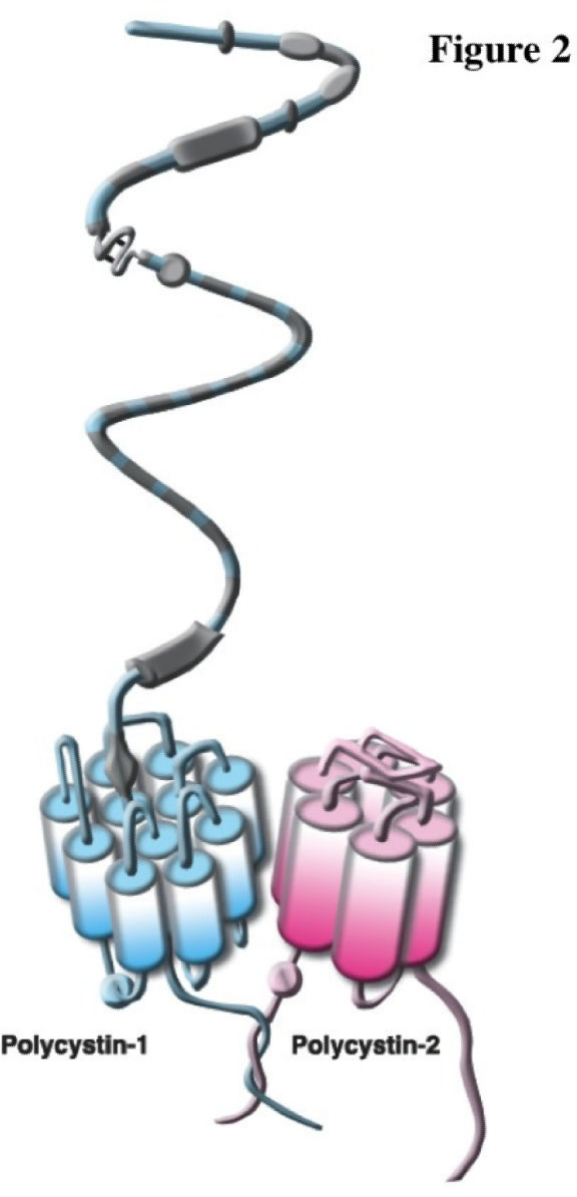
Both polycystin-1 and polycystin-2 form a mechanosensory complex protein through their COOH termini. Polycystin-1 is an eleven-transmembrane protein with a huge extracellular domain, and polycystin-2 is a six-transmembrane calcium channel. There are many other proteins that interact with the intracellular domains of the polycystin complex. This illustration was modified from the original [50].

Polycystin-1 is a large transmembrane protein composed of 4302 amino acids and 11 membrane spanning domains (Figure 2). Polycystin-1 has a long extracellular N terminal domain to mediate mechanosensory function and a short intracellular C-terminus involved in intracellular signaling and interaction with polycystin-2 [51]. Polycystin-1 is expressed in the primary cilia as well as in cell-cell adhesion sites at the basolateral locations like desmosomal junctions and adherence junctions [52,53]. As a signaling entity, ciliary polycystin-1 undergoes several critical functional cleavages, the first of which occurs at a G protein-coupled receptor proteolytic site located at the extracellular N-terminal domain [22]. This cleavage is vital for normal kidney development and polycystin-1 mechanosensory function and signaling [22,54]. The other cleavage site is located at the intracellular C-terminal tail liberating polypeptide fragments that transmit messages to the nucleus and mediate STAT6/P100 [55], AP-1 [56] and canonical Wnt [57] signaling pathways. Fluid-flow is considered an important regulator of these cleavages and contributes to normal function of polycystin-1 [55,56].

Beyond the cleavage of polycystin-1, other signaling pathways of polycystin-1 include polycystin-1 interaction with G-proteins, where polycystin-1may act as atypical GPCR [58,59]. Interestingly, polycystin-1 can activate AP-1 transcription factor and JNK through several heterotrimeric G-proteins [60,61]. AP-1 signaling components are important regulators of cell proliferation and differentiation, which have been implicated in the pathogenesis of ADPKD [62,63]. In addition, polycystin-1 regulates JNK/Bcl-2 apoptosis pathway via Gα_12_ stimulation, an important factor for cyst development [64]. Polycystin-1 also mediates Gαq-activated pathway through calcineurin/NFAT, an important regulator of cell growth, differentiation and adaptation [65]. Another signaling pathway regulated by polycystin-1is mTOR, an important regulator of cell growth. The C-terminal domain of polycystin-1 inhibits the mTOR cascade through the TSC1-TSC2 complex, retarding cell growth [66,67].

Polycystin-2 is a nonselective Ca^2+^ permeable transient receptor potential channel composed of 968 amino acids (Figure 2). Polycystin-2 is an integral protein with six membrane-spanning domains and intracellular C- and N-terminal domains [68]. In addition to its unique subcellular localization in the primary cilia membrane, polycystin-2 is also expressed in the endoplasmic reticulum membrane [69]. Polycystin-2 is involved in calcium signaling through its physical interaction with polycystin-1 [70,71]. It is thus believed that localization of the polycystin-1 and -2 complex in the cilia is required for proper mechanosensory cilia function [72].

The primary cilium in the renal epithelia senses shear-stress resulting from tubular fluid flow, where this mechanical stimulation is processed by the polycystin complex. This complex is essential for mechanosensory function, as revealed in studies using a mutated form of polycystin-1 and blocking antibodies for polycystin-1 [27]. In addition, the presence of primary cilium is essential for the mechanosensory function of the renal epithelium, as revealed in studies utilizing mutated abnormal cilia structure from *Tg737 ^orpk/orpk^* cells and chemical ablation of cilia from ciliated cells [25,73]. In MDCK cells, Praetorius and Spring showed that calcium signal was initiated by a calcium influx, followed by calcium release from IP_3_ sensitive stores [74]. However, Nauli *et al* found that shear stress-induced calcium signal is independent from phospholipase C or IP_3_, instead depending upon ryanodine sensitive stores in embryonic mouse collecting duct epithelial cells [13,27]. To address this discrepancy, Xu et al shows that fluid-shear induced cilia activation can also release ATP in renal epithelia [75]. The ATP will then activate the purinergic signaling pathway, which requires phospholipase C or IP_3_. In addition, the IP_3_ receptor also physically interacts with and is regulated by polycystin-2 in the endoplasmic reticulum membrane, boosting IP_3_- mediated calcium release [76]. On the other hand, polycystin-1 negatively regulates the IP_3_ receptor in the endoplasmic reticulum membrane, creating an opposing effect of polycystin-2 [77].

### 4.2 Chemosensory primary cilia

In IMCD3 cells derived from a kidney collecting duct, an orphan G protein-coupled receptor (GPR88) has been shown to localize to primary cilia [49]. This orphan GPCR plays a modifying role on dopamine-1 and β2 receptors signaling through cAMP. In the proposed model, ciliary GPR88 negatively regulates the human dopamine receptor that is coexpressed and targeted to primary cilia. On the other hand, ciliary targeting of GPR88 protects β2 receptor mediated cAMP activation from its inhibitory effect. As cAMP is known to have an important role in renal pathogenesis, GPR88 might be a therapeutic target.

It is yet unknown whether a purinergic receptor (P2R) is localized to the renal primary cilium, although it is known to localize to the primary cilium of cholangiocyte [37]. It was also shown that only ciliated cells can releases ATP [75,78]. Furthermore, endogenously released or exogenously added ATP enhanced flow-induced calcium signaling, suggesting a chemosensory role of primary cilia. To confirm this observation, ATP scavengers, as well as antagonists for both P2X and P2Y, weaken this cilium-dependent calcium signal. This implies a chemosensory function for renal primary cilia to ATP.

Another proposed chemosensory role of renal primary cilia is attributed to integrins, the extracellular matrix receptors that play an important role in cell adhesion, differentiation and mechanotransduction. Praetorius *et al* showed that β_1_, α_3_ and α_5_ integrins were colocalized to renal primary cilia in MDCK cells [79]. These cells respond to the β_1_ integrin agonist, fibronectin, through eliciting intracellular calcium fluxes. Interestingly, primary cilia potentiate the fibronectin-activated β_1_ integrin-induced calcium signal; however, this pathway is independent of ciliary-mediated flow-induced calcium signaling. This clearly leads to the conclusion of a chemosensory function of the renal primary cilia.

**Figure 3:**
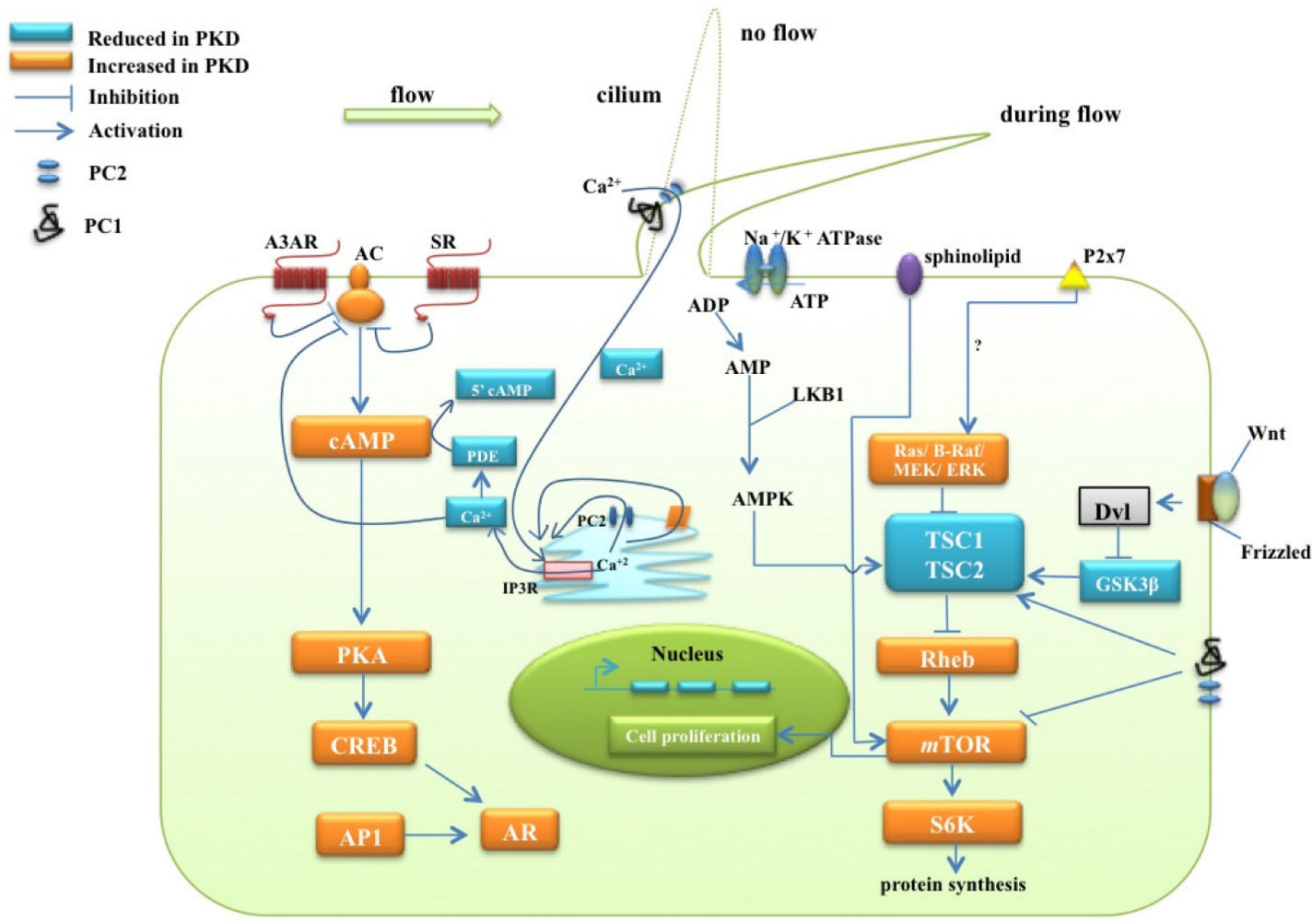
The diagram illustrates the mechanism that polycystin-1 (PC1), polycystin-2 (PC2), signaling proteins, molecules and other receptors exert on signaling pathways leading to cyst formation. The blue box indicates the reduced molecules and signaling proteins in ADKPD. The orange box indicates the increased signaling proteins in ADPKD, which are thought to be responsible for an increase in cell proliferation including cAMP, Ras/Raf/ERK, AC, and mTOR activity. In addition, EGFR activation is also enhanced by amphiregulin (AR) that is abnormally expressed in cystic cells through cAMP, CREB and AP1 signaling (not shown). The sphonigolipid, Na+/K+ ATPase, Wnt and P2x7 purinergic receptors are also involved in the regulation of mTOR and TSC1/TSC2 complex activity. Other receptors that are involved in ADPKD include adenosine receptor-3A (A3AR) and somatostatin receptor (SR), which regulate activity of adenylate cyclase (AC). This illustration was modified from the original [82].

Several lines of evidence revealed a key role of primary cilia in regulating hedgehog (Hh) signaling (Figure 3). In renal cells, among other mammalian cells, Hh signaling function through Smoothened (Smo) and Patched (Ptc) receptors was reported to be essential for cell proliferation, morphogenesis, organogenesis, tissue differentiation and embryonic development. Mutations in IFT proteins, which are essential for cilia structure and function, led to disruptions of Hh pathways and developmental disorders [80]. In the absence of Hh ligands, Ptc is localized to the primary cilia membrane and negatively regulates Hh signaling by repressing Smo [39]. This allows primary cilia to function as chemosensors in response to the Hh ligand. Upon binding to its ligand, Ptc moves out of the cilium, permitting Smo to accumulate in the primary cilium [39,40] and activating the downstream Hh signaling network, mainly through Gli transcription factors [81].

## 5. Vascular Endothelial Function of Cilia

### 5.1 Mechanosensory primary cilia

Primary cilia can also be observed in vascular endothelial cells *in vitro* and *in vivo*. Endothelial primary cilia are relatively shorter than the renal cilia; however, the mechanosensory function of vascular endothelial cells largely resembles that of renal epithelial cells. The ciliary polycystin complex is the most upstream component of the signaling cascade. This complex mediates the translation of extracellular mechanical signals into intracellular biochemical downstream signals, where intracellular calcium is used as an indicator [46].

In addition to the many other vital functions, blood flowing over the endothelia produces an important drag force, also known as shear-stress. Being one of the most important cell-linings within the cardiovascular system, vascular endothelia mechanically sense shear stress and convert it into an array of biochemical signals [16,83]. Endothelial cells can precisely distinguish shear-stress from other types of physical forces imposed on them by blood flow [84]. The significance of blood flowing within the vasculatures is not a new idea. For over 120 years, it has been known that blood vessels develop branches in fast blood flow areas, while branches are not formed in the slow flowing blood vessels of chick embryos. This observation indicates that blood flow velocity and shear stress regulate branching angiogenesis [85,86]. Furthermore, shear-stress has been confirmed to regulate blood vessel diameter, revealing the importance of shear-induced events in vascular growth and remodeling [87,88,89]. Angiogenesis, vessel diameter, vascular growth and remodeling have important implications in health and disease; therefore, it is fundamental to understand the mechanisms and signaling pathways that govern these physiological and pathophysiological processes. Owing to the unique structure, location, length and localization of various functional proteins, primary cilia can be a promising model to illustrate various physiological and pathological processes, in addition to becoming a novel therapeutic target for a mechano-therapy [90].

**Figure 4:**
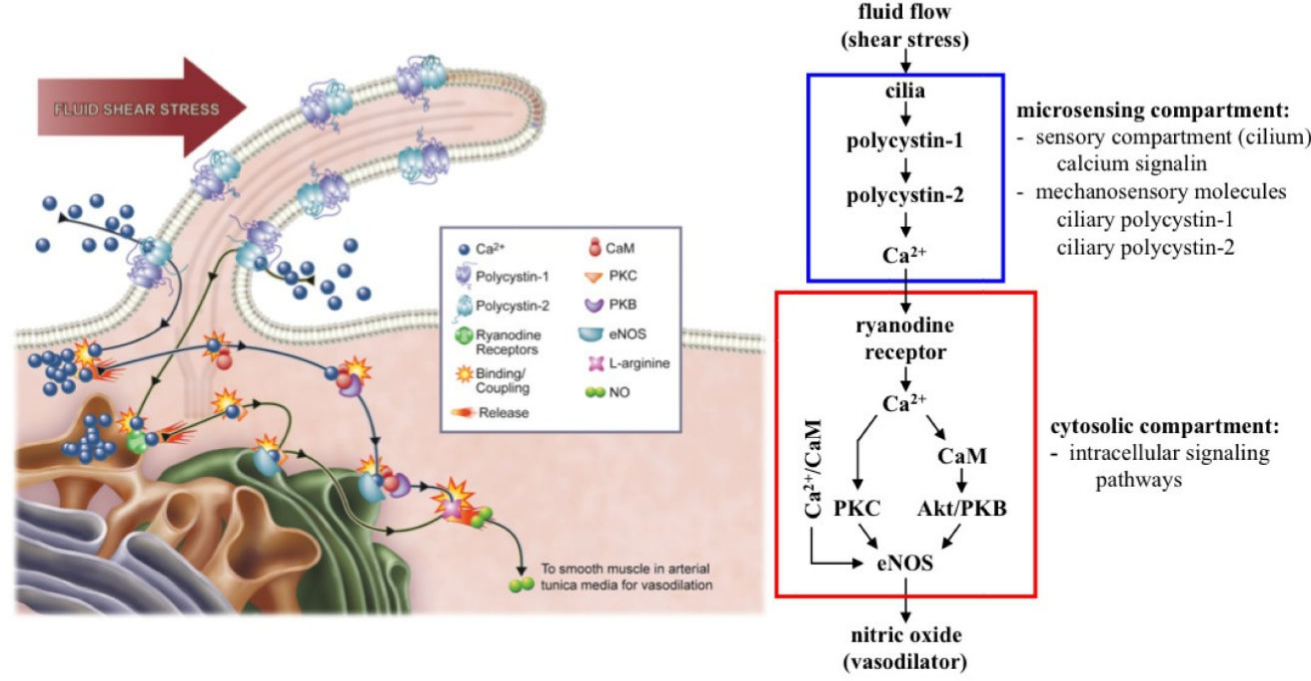
Nitric oxide (NO) synthesis is dependent on the function of endothelial cilia in the vasculature. Primary cilia are sensory organelles that house sensory proteins and function as calcium signaling compartments. The bending of cilia by fluid-shear stress activates the mechanosensory polycystin complex and initiates biochemical synthesis and the release of NO. This biochemical cascade involves extracellular calcium influx, followed by the activation of various calcium-dependent proteins, including calmodulin (CaM), protein kinase C (PKC) and Akt/PKB. This illustration was modified from the original [106].

Endothelial cells detect shear stress via the polycystin-1 and -2 mechanosensory complex localized to primary cilia [28,91]. Primary cilia and polycystin-1 are essential to the mechasosensing capability of an endothelium, as confirmed by Nauli *et al*, who used embryonic aortic endothelial cells with genetic models without polycystin-1 or cilia. Endothelial cells lacking polycystin-1 or cilia are not able to sense fluid-shear stress [91]. AbouAlaiwi et al further showed that ciliary polycystin-2 is essential for endothelial mechanosensory function. Endothelial cells lacking polycystin-2 are insensitive to fluid shear-stress [28]. Collectively, ciliary polycystin-1 first detects mechanical force imposed by blood flow and transfers the signal to polycystin-2 through their C-terminal domain interaction (Figure 2). Polycystin-2 will allow calcium entry into the cell and activate intracellular stores to further release intraorganellar calcium. Extracellular calcium entry is an important event and a prerequisite for the downstream signaling, as confirmed by the inability of endothelial cells to convert mechanical force into intracellular signaling when removing extracellular calcium from the medium [28]. Uprising intracellular calcium ultimately stimulates eNOS, with the resultant production of vasoactive NO. Production of endothelial NO is reported to be dependent upon calcium, calmodulin, PKC and Akt (Figure 4). Endothelial cells with defective cilia structure or function are thus unable to generate NO in response to fluid shear-stress. It is believed that endothelia with defective NO production, in response to shear stress, would result in pathophysiological consequences.

Recently, Hierck et al interestingly observed that endothelial primary cilia are essential for shear stress-induced activation of Krüppel-Like Factor-2 (KLF2) transcription factor [92]. KLF2 can be induced by high levels of shear-stress and repressed by low and disturbed flow. Thus, KLF2 is considered a shear-stress marker [93]. Being an important regulator of vasculature status at the transcription level, KLF2 can induce eNOS and thrombomodulin, while it represses endothelin, angiotensin converting enzyme, and proinflammatory and anti-fibrinolytic genes transcription [94,95,96]. Hence, KLF2 plays an important protective role and is a hemodynamic regulator required for proper cardiovascular development and function. Thus, endothelial primary cilia, through the KLF2 pathway, might have promising therapeutic implications.

The mechanosensory endothelial primary cilia also play a key role in autoregulating their own structure and function, as well as in regulating cellular structure and function integrity. As an autoregulatory organelle, the structure and function of the mechanosensory endothelial primary cilia are regulated by the dopamine receptor, which is localized to the primary cilium *in vitro* and *in vivo* [34,97]. Abdul-Majeed and Nauli found that activation of the ciliary dopamine receptor results in elongation of cilia, with the concomitant enhancement of ciliary mechanosensory function [34]. Enhanced mechanosensory function is reported to be mediated through actin differentiation and cofilin dephosphorylation in wild type cells, while it is distressed in cilia mutant endothelial cells. Interestingly, defective mechanosensory function in mutant cells can be restored by ciliary dopamine receptor activation [34]. Most recently, it was further shown that PDGFRα signaling in the primary cilium regulates NHE1-dependent fibroblast migration via coordinated differential activity of MEK1/2-ERK1/2-p90RSK and AKT signaling pathways [98].

The role of the primary cilium in regulating whole cell integrity and function further reveals cytoskeleton orientation as an indicator [34]. Jones et al also reported that an intact functional cilium is required for actin cytoskeleton organization, directional migration and barrier permeability in the endothelium [45]. Endothelial cells with defective structure or function of cilia exhibit reduced actin stress fibers and focal adhesions, resulting in impaired directional migration and high apico-basal permeability. These events are proposed to be mediated in part through hsp27, which was found suppressed in the mutant endothelial cells. Collectively, these results verify the importance of primary cilia and sensory polycystins complex in cytoskeleton organization.

The significance of functional mechanosensory endothelial primary cilia on cell division has also been studied. AbouAlaiwi et al show that structurally and functionally intact endothelial primary cilia are essential for proper cell division [99,100]. Defective primary cilia structure or function shows multipolar spindle formation, mitotic abnormality, centrosomal amplification and cell polyploidy. These cell division abnormalities in the mutant cells are reported to be mediated through abnormally suppressed expression of survivin, a chromosomal passenger. These findings might provide some hints about the pathophysiological pathways of aberrant cell proliferation in cystic kidneys as well as in blood vessel aneurysms associated with ADPKD and support the importance of the role of primary cilia in the disease [101].

The relationship between mechanosensory endothelial primary cilia and atherosclerosis is a vital perspective with direct clinical consequences. In relation to shear stress, atherosclerosis is generated in areas where endothelial cells are exposed to turbulent blood flow and are found at bifurcations of blood vessels. On the other hand, high shear-stress and laminar blood flow can retard atherosclerosis formation in these areas, proposing a vital role for shear-stress in this pathological process [102]. In studies using adult mouse aortic arch and common carotid arteries, primary cilia are found at the atherosclerotic predilection sites [103]. Furthermore, in various experimentally induced flow patterns, primary cilia are found expressed in areas of low or turbulent shear stress. Supporting these findings, primary cilia are expressed in atheromatous plaques of adult human aortic endothelial cells more than in the non-affected areas or fibrous plaque areas [104]. It was also reported that primary cilia disassembled after 2 hours of continuous laminar high shear stress with the termination of IFT in cultured human umbilical vein endothelial cells [105]. These studies suggest a role for endothelial primary cilia as mechanosensors in endothelial dysfunction and consequently in atherogenesis at various vascular sites.

### 5.2 Chemosensory primary cilia

In addition to its mechanosensory function, the primary cilium gains some attention as a chemosensor. To be a chemosensor, endothelial primary cilium should be a host for a functional receptor that mediates distinct downstream signaling cascades when binding to a ligand. Dopamine receptor type-5 (DR5) is a D1-like dopaminergic receptor. Abdul-Majeed and Nauli found that DR5 localizes to primary cilia of cultured mouse embryonic aortic endothelial cells *in vitro* and mouse femoral arteries *in vivo* [34]. By binding to its ligand, ciliary DR5 triggers downstream signaling manifested by increasing intracellular calcium. In addition to calcium, dopamine and many other chemical activators and inhibitors also evoke endothelial cilia.

Teilmann and Christensen reported the presence of primary cilia in ovarian and extraovarian tissues, including endothelial cells of the female mouse reproductive system [107]. They found that angiopoietin receptors Tie1 and Tie2, receptor tyrosine kinases, are localized to the primary cilia of ovarian endothelial cells in mice. Upon binding their ligands, angiopoietins, these receptors play a vital role in vascularization through VEGF [108] and endothelial apoptosis through the PI3K and Akt pathways [109]. These studies provide another attribute to endothelial primary cilia as chemosensory organelles.

## 6. Conclusion

More evidence has emerged to support the important roles of primary cilia play in disease and development. It is now known that primary cilia function as mechanosensory and chemosensory organelles. However, any defect in primary cilia can trigger a wide range of complications, such as in the kidney, vasculatures and many other organs. Yet, many more ciliary proteins involved in either mechanosensory or chemosensory function are still to be sorted out. Only through thorough understanding of individual molecules within the sensory cilia can we appreciate the complexity of the primary cilium in its very diverse roles.
